# Extracting coherent tree-ring climatic signals across spatial scales from extensive forest inventory data

**DOI:** 10.1371/journal.pone.0189444

**Published:** 2017-12-27

**Authors:** Louis Duchesne, Loïc D’Orangeville, Rock Ouimet, Daniel Houle, Daniel Kneeshaw

**Affiliations:** 1 Direction de la Recherche Forestière, Ministère des Forêts, de la Faune et des Parcs du Québec, Einstein, Quebec City, Quebec, Canada; 2 Centre d’Étude de la Forêt, Université du Québec à Montréal, Case Postale, Succursale Centre-Ville, Montreal, Quebec, Canada; 3 Department of Biology, Indiana University, Bloomington, IN, United States; 4 Consortium sur la Climatologie Régionale et l’Adaptation aux Changements Climatiques (Ouranos), Montreal, Quebec, Canada; Pacific Northwest National Laboratory, UNITED STATES

## Abstract

Increasing access to extensively replicated and broadly distributed tree-ring collections has led to a greater use of these large data sets to investigate climate forcing on tree growth. However, the number of chronologies added to large accessible databases is declining and few are updated, while chronologies are often sparsely distributed and are more representative of marginal growing environments. On the other hand, National Forest Inventories (NFI), although poorly replicated at the plot level as compared to classic dendrochronological sampling, contain a large amount of tree-ring data with high spatial density designed to be spatially representative of the forest cover. We propose an *a posteriori* approach to validating tree-ring measurements and dating, selecting individual tree-ring width time series, and building average chronologies at various spatial scales based on an extensive collection of ring width measurements of nearly 94,000 black spruce trees distributed over a wide area and collected as part of the NFI in the province of Quebec, Canada. Our results show that reliable signals may be derived at various spatial scales (from 37 to 583,000 km^2^) from NFI increment core samples. Signals from independently built chronologies are spatially coherent with each other and well-correlated with independent reference chronologies built at the stand level. We thus conclude that tree-ring data from NFIs provide an extraordinary opportunity to strengthen the spatial and temporal coverage of tree-ring data and to improve coordination with other contemporary measurements of forest growth to provide a better understanding of tree growth-climate relationships over broad spatial scales.

## Introduction

Because tree-ring measurements can be used to estimate the influence of environmental forces on annual wood production [1,2], they have been recognized as a key resource to provide a long-term perspective of the growth response to global environmental changes [3,4]. According to the principle of aggregate tree growth, tree-ring series contain information about a combination of drivers, including not only climate, but also age-related growth trends, soil conditions, disturbances, inter-tree competition, and random processes, working at different spatial scales.

The traditional dendrochronological approach consists of building annually dated tree-ring chronologies, either to reconstruct climate variables (dendroclimatology) or to date and analyze disturbances and variations in the local environment (dendroecology) [5]. Quality control of ring width measurements and dating (crossdating) is usually performed to ensure proper identification of the exact years of formation of annual rings [6]. Raw ring width time series of a given population are typically standardized, i.e. long-term growth trends are removed to isolate interannual changes, and averaged into a single master chronology representative of the local signal. Averaging tree-ring series at the stand scale minimizes tree-scale noise, although the residual growth signal still contains stand-scale noise from local topography, soil conditions, inter-tree competition, and local disturbances, in addition to climate [7,8].

In recent decades, broad-scale networks of tree-ring measurements have allowed relationships between species-specific growth and thermal and moisture variations to be studied at the local, regional, continental, or near-hemispheric scales [9]. Averaging ring width time series over broad spatial scales provides a better assessment of the commonly shared interannual growth patterns, and reduces tree- and stand-scale noise [9]. It has indeed been demonstrated that the accuracy of regional chronologies and the assessment of climate-growth relationship tend to improve more by increasing the number of plots rather than the number of trees per plot [10]. Consequently, broad-scale tree-ring networks could increase our capacity to estimate species-specific climate sensitivity [11] and to predict forest responses to environmental changes at regional scales [12], while providing more robustness for broad-scale climate reconstructions [13]. For this reason, multiple regional-scale networks of tree-ring measurements have been established over the last decade [9,14], the rationale being that tree-ring records from networks covering broad areas should be representative of commonly shared growth patterns as the weight of local, non-climatic factors is reduced [9,10,15]. Furthermore, the spatial coherence of independent tree-ring chronologies from broad areas thus plausibly reflects broad-scale climate forcing [16].

Most broad-scale dendroclimatic studies produced over the past few decades have made extensive use of the International Tree-Ring Data Bank (ITRDB), the largest open-access tree-ring archive managed by the U.S. National Oceanic and Atmospheric Administration [4,17,18]. The database currently includes tree-ring width chronologies from 4,467 studies. However, few chronologies have been updated and the number of new chronologies being added is declining. Thus, more than three quarters of the ITRDB chronologies do not extend beyond the year 2000 [4]. This and the lack of spatial representativity could have negative consequences for paleoclimatic investigations, in addition to precluding the integration of tree-ring width data with the increasingly available data from instrumental weather stations, remote sensing, national forest inventories, and other *in situ* measurements of forest growth [4,19]. Moreover, tree-ring data are mostly distributed in North America and Europe, focusing on marginal growing environments selected to maximize the climatic signal, and the selective sampling of the biggest trees within populations (with the aim of producing long chronologies) often leads to bias in archived datasets. This further limits their usability for dendroecological investigations and for documenting the global forest response to contemporary environmental changes [4,18,20,21].

Recent studies demonstrate that rudimentary increment core sampling (i.e. sampling with low replication at the stand level) from National Forest Inventories (NFI) may potentially be used for dendroecological investigations [22–31]. Indeed, although tree-ring sampling is rudimentary or non-existent in some NFIs [4], many NFIs include increment core samples that were taken to age stands and determine site index, as well as to estimate annual average tree growth [23,26–28,31].

Increment core samples from NFIs are typically poorly replicated at the plot level as compared to classic dendrochronological sampling, which limits the possibility of crossdating validation at the plot scale. Although crossdating is not a prerequisite for forest inventories (i.e. tree age and site index estimation) [26], the presence of a significant proportion of poorly crossdated chronologies in a dataset could violate one of the fundamental principles of dendrochronology and thus seriously jeopardize the potential of the NFI dataset for any study requiring knowledge of the exact year of formation of annual rings in increment cores [32]. Considering the huge effort invested in the sampling and processing of NFI tree-ring databases, it would, however, be unfortunate if the scientific community were unable to use these tree-ring data because of the above-mentioned issue. On the other hand, NFIs have much higher spatial densities and are generally designed to be geographically unbiased and spatially representative of the forest cover [23,25,26]. Developing a procedure that would permit researchers to extract a reliable dendroclimatic signal from NFI tree-ring data would provide an extraordinary opportunity to strengthen the spatial coverage of tree-ring width data in terms of climate zone, species composition, and forest productivity, and to increase coordination with long-term monitoring plots in NFIs [4,26].

In this study, we assessed whether reliable and spatially coherent signals can be extracted from an extensive collection of tree-ring width measurements from 94,000 black spruce trees sampled in 34,000 stands distributed across 583,000 km^2^ in Quebec, Canada. Because climatic factors are less spatially heterogeneous than non-climatic factors, we hypothesized that (i) reliable tree-ring signals may be assessed at various spatial scales, but that the amount of covariance within tree-ring signals would be negatively correlated with geographic distance among trees, and (ii) for a given spatial scale of tree-ring width series aggregation, coherency among master chronologies will decrease with geographical distance.

## Methods

### Study area

Historical forest inventory programs in Quebec, Canada, were mainly conducted below the current northern limit for forest management, which extends from latitude 45° to 52°N and covers approximately 583,000 km^2^, of which 434,667 km^2^ are classified as productive forest, i.e. more than 30 m^3^ ha^-1^ of wood produced in 120 years. This territory is characterized by three different forest subzones from South to North: hardwood forest, temperate mixed-wood forest and continuous boreal forest. The temperate mixed-wood forest (latitude 47° to 48°N) marks the transition between the hardwood forest to the south, which is dominated by sugar maple (*Acer saccharum* Marsh.), and the coniferous forest to the north, which is dominated by balsam fir (*Abies balsamea* (L.) Mill.) and black spruce (*Picea mariana* (Mill.) B.S.P.). Normal mean annual temperatures (1971–2000) range from approximately -4.7 ^o^C to 6.7 ^o^C while annual precipitation ranges from approximately 700 mm to 1600 mm. At the beginning of the 20^th^ century, logging was confined to areas south of latitude 49°N, but now extends up to latitude 51°N. Along with forest management, fire and spruce budworm (*Choristoneura fumiferana* Clemens) outbreaks are the main broad-scale disturbances regulating boreal forest dynamics [33].

### Data collection

**Fig 1:**
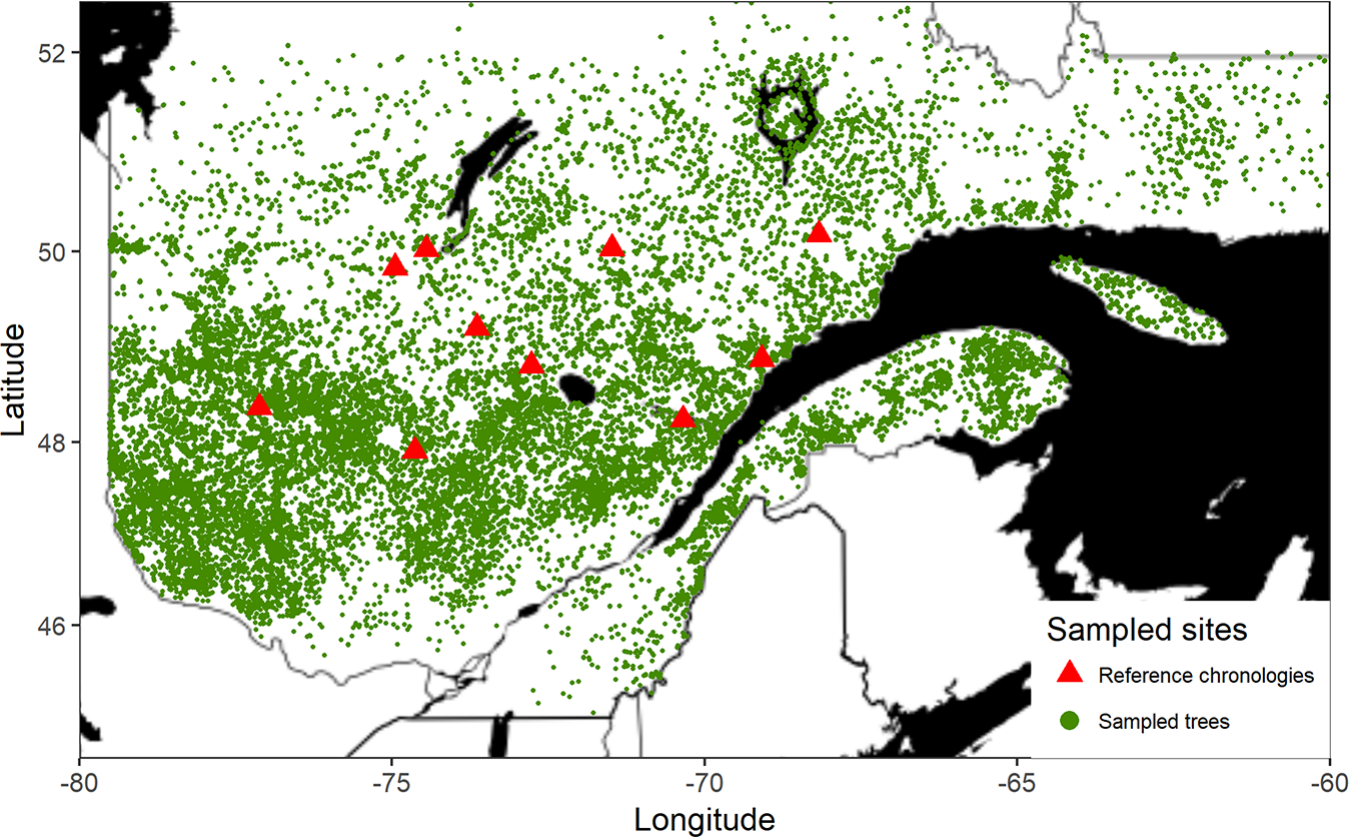
Map showing the location of tree-ring sample sites. Red triangles show the locations of the ten reference chronologies while green points show the locations where cores of black spruce trees were sampled in national forest inventories in Quebec, Canada.

Forest inventory programs conducted by provincial forest authorities use temporary and permanent sample plots to comprehensively characterize existing forest resources. The forest inventory is based on a stratified randomized sampling design with proportional allocation. Forest stands interpreted from aerial photographs were first stratified based on stand characteristics (composition, density, height, age), edaphic properties (slope, drainage, deposit), and disturbance history [34]. Circular plots (radius = 11.28 m, area = 400 m^2^) were then proportionally allocated to each stratum according to their respective surface area [35]. In each plot, the diameter and visual health of every tree, the composition of the understory and soil thickness, deposit type, and drainage class were recorded. Increment cores were collected in both permanent and temporary sample plots according to strict sampling protocols [35,36]. In each temporary sample plot, three trees (diameter at breast height (DBH) > 90 mm) were cored at one meter above ground: one was selected randomly, another was selected randomly among the four biggest trees (in DBH) of the dominant species, and the third had a diameter closest to the mean diameter of the dominant tree species. In permanent sample plots, up to nine trees were cored: five were selected randomly; two were selected randomly among the four biggest trees (in DBH) of the dominant species; one had a diameter close to the mean diameter of the dominant tree species; and the last had a basal area at breast height closest to the 30^th^ percentile of the distribution of stem basal area for the dominant species. The current study uses complete increment core data for black spruce trees, collected from 34,105 permanent and temporary sample plots, for a total of 94,120 black spruce trees (Fig 1). Approximately 90% of the sampled black spruce stands contain 3 tree core samples or less and only 0.16% of the sampled sites contain 10 or 11 black spruce samples.

### Laboratory analysis

Cores were dried, glued to a wooden holder, and sanded successively with 120, 220 and 320 grit sandpaper to obtain a smooth surface. Ring boundaries were first carefully detected and identified under binocular magnification by specialized technicians, then measured to the nearest 0.01 mm with the WinDendro Image Analysis System for tree-ring measurement (Regent Instruments Inc.) after scanning the sample at a resolution of 1000 dots per inch. A calendar year was attributed to each ring, the outermost ring corresponding to the year of tree sampling, or exceptionally to the year before for plots sampled prior to the start of tree-ring formation.

### Tree-ring data validation and chronology computation

It is well known that obtaining tree-ring data is costly and time-consuming [23,26]. Due to the substantial number of samples processed (94,000 cores) and the low sample replication at the stand level (one to eleven trees of the same species per plot, i.e. depends on stand and region level tree diversity), tree-ring width measurements were carefully dated but their crossdating was not validated at the stand level according to the standard procedure before archiving the information in the database.

Dendrochronologists generally use computer-assisted quality control of tree-ring dating and measurements. COFECHA is the most commonly used program for that purpose [37]. In the absence of an established reference chronology for a given region and species, the standard procedure consists of removing low-frequency variations, averaging data to form a master dating series, and correlating individual transformed series with the master dating series [37]. The program can thus be used to accept or reject a series for inclusion in a site chronology [37]. We used a similar procedure (described below) to apply an *a posteriori* validation procedure to correctly select individual tree chronologies and extract reliable signals at various spatial scales from Quebec’s extensive NFI tree-ring database.

**Fig 2:**
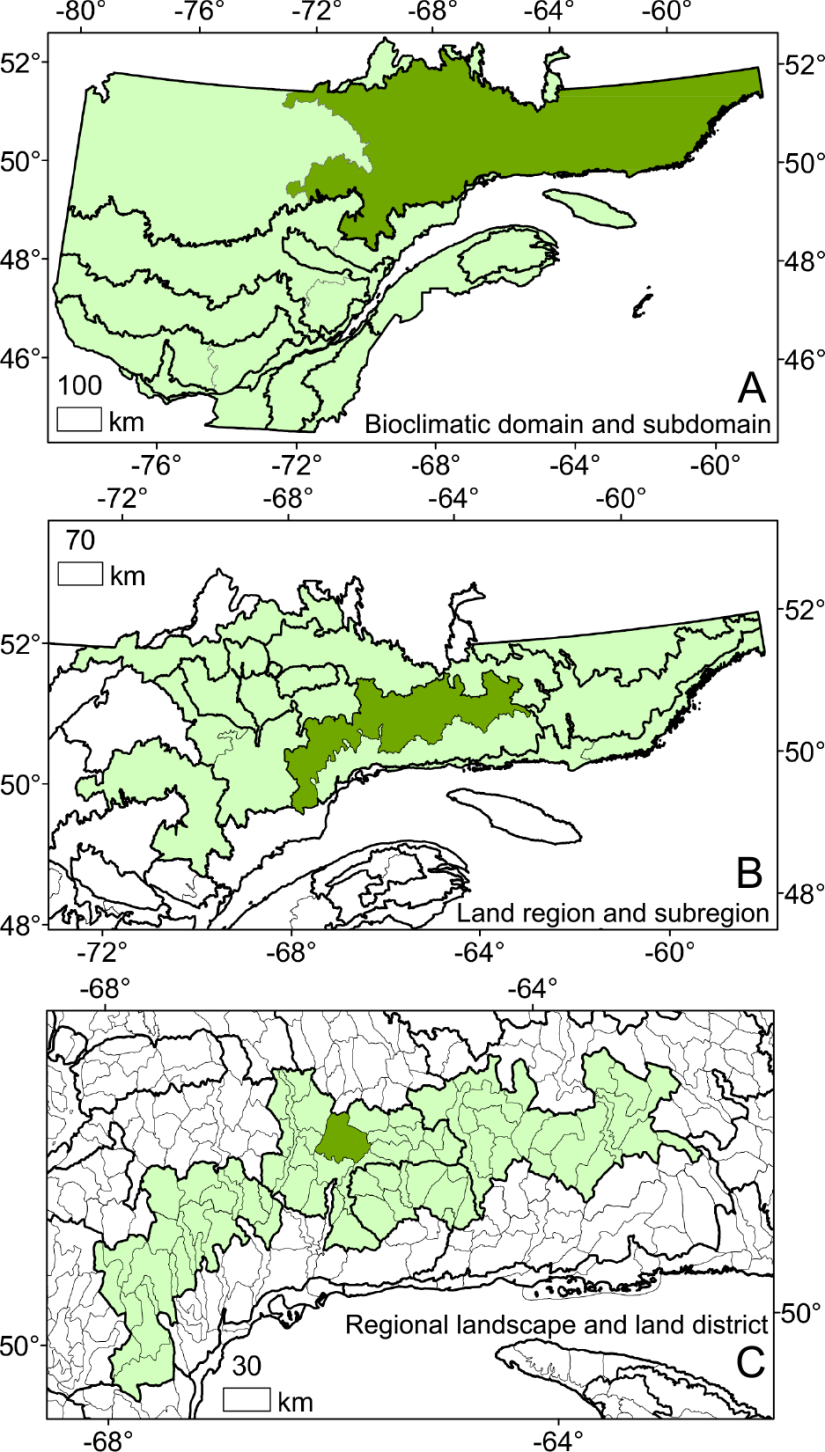
Example of the hierarchical structure of the forest Land Hierarchical Classification System (LHCS) developed by the Quebec Ministry of Forest, Wildlife and Parks. The provincial territory is separated into 12 bioclimatic domains (thick lines) and sub-domains (thin lines) (A). Each of them is separated according to land region (thick lines) and sub-region (thin lines) (B), these latter being separated into landscape units (thick lines) and ecological districts (thin lines) (C).

The most common approach for analyzing broad-scale tree-ring signals is to group trees cored from nearby plots and to build a regional chronology for each climatically homogeneous zone defined within the study area [10]. In this study, trees were grouped based on the similarity and proximity of their environments to generate master chronologies at various spatial scales using the Land Hierarchical Classification System (LHCS) developed by the Quebec Ministry of Forest, Wildlife and Parks [38]. The LHCS organizes the territory into units similar in climate, vegetation, or geomorphology at 11 nested spatial scales. At the finest scale, the territory is separated into 2,545 ecological districts (median area: 228 km^2^), grouped into 185 landscape units (median area: 3,165 km^2^), grouped into 71 sub-regions (median area: 8,726 km^2^) (Fig 2). Because the LHCS does not explicitly take into account the local soil physical environment, we also grouped sites by soil physical environmental types (nested to the LHCS) according to soil type (mineral, organic), moisture regime, and texture as determined during field sampling (Table 1).

**Table 1:** Schematic classification of soil physical environments described in Quebec.

	Mineral soil				Organic soil
Soil moisture regime	Very shallow (<25 cm) or very stony	Coarse texture	Medium texture	Fine texture	
Xeric to mesic		1	2	3	
Hygric	0	4	5	6	
Hydric			7		8 –Fen 9 –Bog

**Xeric**—Dry, little moisture retention, excessively drained. Water removed very rapidly in relation to supply; soil is moist for brief periods following precipitation.

**Mesic**—Moist, adequate soil moisture retention year-round. Water removed somewhat slowly in relation to supply; soil may remain moist for a significant, but sometimes short, period of the year. Available soil moisture reflects climatic inputs.

**Hygric**—Water removed slowly enough to keep soil wet for most of the growing season; permanent seepage and mottling; gleyed (greenish-blue-grey) mottles common in the soil profile.

**Hydric**—Wet; periodically or often flooded by water. Water removed so slowly that water table is at or above soil surface all year; gleyed mineral or organic soils.

The study was conducted for the 1900–2012 period. In order to emphasize year-to-year variability in tree-ring series and to remove non-climatic trends due to tree age, stem size, stand dynamics, as well as mid-term trends in climate, we fitted each individual series with a cubic smoothing spline with a 50%-frequency cut-off at ten years [39]. We used a highly flexible spline function to remove most of the low-frequency variations and focus on high-frequency variations mainly associated with interannual climate variability. Ring-width measurements were transformed into dimensionless indices by dividing raw values with spline function estimates. Because the autocorrelative structure present in tree-ring series can blur the climatic signal and interfere with several statistical assumptions, autoregressive models of various orders were fitted to each standardized tree-ring series, the best one was selected using the Akaike Information Criterion [40], and the residual series were retained for the study [41].

Spearman correlation coefficients were computed between each individual standardized tree-ring series of a given spatial unit and its corresponding average chronology using bi-weighted robust estimates of the means of all standardized series [41]. Individual series that were significantly correlated (p < 0.05) to this “raw” mean regional chronology were used to compute a new “refined” average regional chronology. Spearman correlation coefficients were again computed between each individual standardized tree-ring series and this refined average regional chronology. Here again, significantly correlated series were used to recompute the refined regional chronology. Finally, Spearman correlation coefficients were computed between all individuals composing that regional chronology and the refined chronology itself, and only significantly correlated series were kept building a refined regional chronology. This last step was iteratively repeated until all individual tree samples included in the regional chronologies were significantly correlated to it (p < 0.05). In comparison with our approach, COFECHA used a threshold probability value of 0.01 to underline and flag possible problems in tree-ring width series. The processing of raw tree-ring width measurements was completed using the “dplR” package [42] in the R software [43]. Regional chronologies were considered valid when they displayed an expressed population signal (EPS) value ≥ 0.85. While frequently misinterpreted as an indicator of chronology suitability for climate reconstruction purposes, EPS is a measure of how well the chronology signal is a good estimator of the population signal [44,45]. The statistic estimates the strength of the common signal relative to the total signal (common signal + noise) in a tree-ring time series [44].

### Probability of false-positive selection and possible hidden signals

Autocorrelative structure is inherent in individual tree-ring series. As a result, improperly dated individual series can still be significantly correlated to the average chronology, increasing the probability of false-positive selection. As previously mentioned, autoregressive structure was removed from individual standardized series. To explore the probability of false-positive selection associated to the remaining autocorrelative structure in standardized series, the population distribution of first-order autocorrelation has been documented.

In order to verify that the rejected series did not contain any additional hidden signals, we applied our selection procedure to the rejected data for each spatial unit and scale. A principal component analysis (PCA) was also computed on the scaled matrix of all residual series in each spatial unit for each spatial scale to ensure that the selected residual series represent the dominant signal in the group of series.

As performed by the COFECHA program, to allow detection of dating error, rejected standardized series have been tested for correlation, segment by segment, against their respective regional chronologies built at the landscape scale. This has been performed by calculating correlations for each segment of the series under examination with the regional chronology at the point of dating, and also at each position from 5 years earlier (-5) to 5 years later (+5) [37]. Segment tested are 22 years long (1944–1965, 1955–1976, 1966–1987), restricting the analyses to the 1944–1987 period.

### Reliability of the computed chronologies and coherence across space

Reliability of the computed chronologies at various spatial scales was assessed by computing several descriptive statistics, including the mean correlation of individual residual series with their corresponding master chronologies (Mcor), the mean correlation between all pairs of residual series (Rbar), and the EPS [44,46].

We assessed spatial coherency among master chronologies by analyzing the spatial autocorrelation between independently built chronologies at each spatial scale. Correlations (Spearman's rho) were expressed as a function of distance (i.e. correlograms) considering the centroid of the spatial unit as the reference point. Similarly, we assessed spatial coherency among mean annual temperature and yearly precipitation time series. We simulated temperature and precipitation (1901–2012) for each centroid of the spatial unit with the BioSIM stochastic weather generator [47]. BIOSIM provides forecasts based on regional air temperature and precipitation, interpolated from nearby weather stations and adjusted for elevation and location differentials with regional gradients. To remove long-term trends and isolate interannual changes, climate time series were standardized using the same procedure as used for tree-ring time series.

We also assessed the coherency of master chronologies by analyzing correlations between our chronologies and classic neighbouring reference chronologies built at the stand scale. For the area under study, nine black spruce reference chronologies from the International Tree-Ring Data Bank (ITRDB) and an additional seven black spruce reference chronologies from the *Réseau d’étude et de surveillance des écosystèmes forestiers* (RESEF) monitoring network [48] were available. From the nine ITRDB chronologies, only four included the raw tree-ring series, allowing us to perform a uniform data standardization. Out of these four ITRDB chronologies, one displayed EPS values below 0.85 for the 1900–1988 period, and was thus rejected. The remaining ten chronologies (three from the ITRDB and seven from the RESEF) are well distributed spatially (Fig 1), contain 24 to 50 trees, and display mean correlations to the master chronology, EPS value and Rbar of 0.62 (range 0.52–0.73), 0.95 (range 0.91–0.98) and 0.43 (range 0.31–0.55), respectively (Table 2). We assessed the coherency of new master chronologies by calculating Spearman correlations between independently built chronologies and the ten neighbouring classic reference chronologies at the two finest spatial scales (district and landscape). We limited the comparison to chronologies located within a 40-km radius (district) and 80-km scale (landscape). The extent of the radius was determined as a reasonable trade-off between spatial proximity and the number of chronologies.

**Table 2:** Characteristics of the reference crossdated black spruce chronologies used to validate the newly formed chronologies.

Name	Source	Location	N trees	N cores	Range used	EPS	Rbar	Mcor
Ref054	ITRDB	50.03, -71.48	24	2	1905–1988	0.932	0.365	0.583
Ref055	ITRDB	50.17, -68.17	26	2	1912–1988	0.925	0.335	0.567
Ref075	ITRDB	50.02, -74.45	24	2	1901–1988	0.905	0.314	0.518
Ref202	RESEF	48.24, -70.35	45	2	1923–1996	0.959	0.434	0.641
Ref203	RESEF	48.81, -72.77	39	2	1923–1995	0.963	0.418	0.628
Ref204	RESEF	49.21, -73.65	30	2	1941–1996	0.965	0.506	0.649
Ref404	RESEF	47.90, -74.63	41	2	1941–1996	0.978	0.547	0.660
Ref801	RESEF	48.37, -77.12	49	2	1940–1997	0.977	0.488	0.664
Ref902	RESEF	48.88, -69.08	50	2	1932–1995	0.982	0.549	0.733
Ref1001	RESEF	49.83, -74.96	48	2	1942–1996	0.962	0.369	0.577

Note: Mcor, mean correlation of individual residual series with their corresponding master chronologies; Rbar, mean correlation between all pairs of residual series; EPS, expressed population signal.

Overall, our validation process is similar to the framework used for building the Forest Inventory and Analysis (FIA) tree-ring data set in the U.S. where quality control was conducted by comparing chronologies with the closest available public chronology from the ITRDB and with unpublished chronologies in gap areas, and by using the COFECHA program to assess the quality of measurements and dating [26].

## Results

### Reliability of the computed chronologies

Depending on the spatial scale considered, our iterative selection procedure retained 35.7% to 73.2% of all available samples, and 60.7% to 81.2% of all sites, the number and proportion of selected trees and sites (inventory plots) were decreasing as the grouping area increased (Table 3). As compared to the initial selection with the iterative procedure, comparable sample selection ratios were observed when rejecting chronologies with EPS values lower than 0.85, except for the finest spatial scale. In those cases, the selection ratio was considerably lowered by the EPS criteria due to the low sample frequencies per spatial unit (Table 3). Using the soil physical environment for grouping slightly increased the number of sites and trees retained by the iterative procedure and slightly improved the quality of the valid chronologies (EPS, Mcor, and Rbar, Table 3). It might have been interesting to detail whether there were differences in chronologies among soil physical environment types, but this analysis was beyond the scope of the study. The number of valid chronologies generated also depended on the spatial scale considered, with one chronology covering 100% of the territory being generated at the meridional scale and 562 chronologies covering 35% of the territory being generated at the smallest, district, scale. Their reliability, as indicated by Mcor and Rbar statistics, generally decreases as the grouping area increases.

**Table 3:** Summary of the selection process leading to the formation of valid black spruce chronologies (EPS ≥ 0.85, 1900–2012) from the broadest to the finest spatial scales with and without considerations of the soil physical environment type (+soil).

Spatial scale	Median area (km^2^)	Selected series with iterative procedure		Selected series with chronology EPS ≥ 0.85		Valid chronological statistics*				
		Sites (%)	Trees (%)	Sites (%)	Trees (%)	N	Covered area (%)	Mean series frequency in chronology [range]	Mean correlation to master chronology [range]	Rbar [range]
**Meridional**	583,077	20,709 (60.7)	33,634 (35.7)	20,709 (60.7)	33,634 (35.7)	1	100	33,634	0.34	0.12
+ soil	-	21,228 (62.2)	35,153 (37.4)	21,228 (62.2)	35,153 (37.4)	10	100	2,318 [1,131–13,314]	0.35 [0.34–0.39]	0.13 [0.11–0.15]
**Domain**	81,193	21,820 (64.0)	37,384 (39.7)	21,817 (64.0)	37,377 (39.7)	5	97.8	8,006 [167–13,200]	0.37 [0.32–0.40]	0.15 [0.11–0.17]
+ soil	-	22,517 (66.0)	39,354 (41.8)	22,418 (65.7)	39,181 (41.6)	40	97.8	644 [46–6 595]	0.38[0.32–0.47]	0.15 [0.10–0.20]
**Sub-domain**	39,798	23,565 (69.1)	42,164 (44.8)	23,554 (69.1)	42,142 (44.8)	9	96.8	4,947 [156–10,495]	0.38 [0.33–0.40]	0.17 [0.12–0.20]
+ soil	-	24,276 (71.2)	44,039 (46.8)	24,088 (70.6)	43,707 (46.4)	72	96.8	372 [41–4,869]	0.39 [0.32–0.46]	0.17 [0.09–0.24]
**Region**	11,562	25,436 (74.6)	47,461 (50.4)	25,373 (74.4)	47,350 (50.3)	41	94.7	771 [92–4,096]	0.38 [0.31–0.43]	0.16 [0.10–0.22]
+ soil	-	26,039 (76.4)	49,939 (53.1)	24,475 (71.8)	46,353 (49.2)	190	94.7	142 [30–1,794]	0.40 [0.31–0.48]	0.18 [0.09–0.27]
**Sub-region**	6,589	25,568 (75.0)	48,040 (51.0)	25,466 (74.7)	47,815 (50.8)	59	93.5	519 [49–3,903]	0.40 [0.31–0.47]	0.17 [0.10–0.27]
+ soil	-	26,314 (77.2)	51,105 (54.3)	23,959 (70.3)	45,861 (48.7)	228	89.5	124 [29–1,794]	0.40 [0.31–0.51]	0.18 [0.09–0.32]
**Landscape**	2,559	26,449 (77.6)	51,123 (54.3)	26,169 (76.7)	50,492 (53.7)	146	88.9	263 [49–1,598]	0.40 [0.31–0.47]	0.18 [0.10–0.30]
+ soil	-	27,494 (80.6)	56,040 (70.2)	21,409 (62.8)	42,151 (44.8)	366	81.1	82 [7–642]	0.42 [0.29–0.70]	0.19 [0.08–0.46]
**District**	203	28,841 (84.6)	61,950 (65.8)	18,744 (55.0)	38,640 (41.1)	562	34.9	59 [18–608]	0.43 [0.31–0.55]	0.20 [0.09–0.41]
+ soil	-	27,698 (81.2)	68,938 (73.2)	6,702 (19.7)	14,586 (15.5)	288	16.2	45 [7–294]	0.45 [0.33–0.70]	0.21 [0.09–0.46]

* All statistics are medians of the population.

Rbar, mean correlation between all pairs of residual series.

EPS, expressed population signal.

### Signal coherence across space and with neighbouring reference chronologies

**Fig 3:**
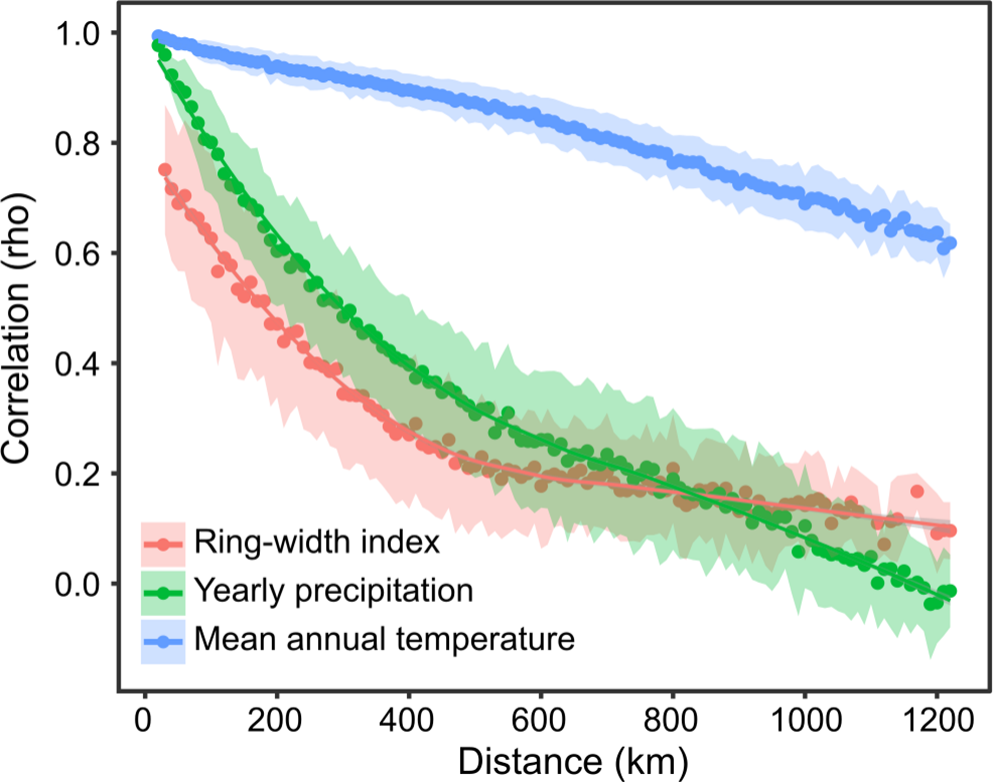
Average effect of geographical distance on the similarity between tree-ring width index, yearly precipitation and mean annual temperature times series built at the landscape scale. Spearman correlations, averaged per binned distance (30-km intervals), were used to compare chronologies. The blue area represents the standard deviation from the means. The exponentially decreasing pattern is identical across all spatial scales.

The distance correlogram computed from correlations between tree-ring and climate chronologies displays a strong spatial autocorrelation (Fig 3). The negative effect of distance on the common signal between master chronologies can be described as non-linear exponential decay, with the highest rate of coherence loss at short distances, from rho = 0.75±0.12 between 30 km-distant populations to rho = 0.51±0.13 between 180 km-distant populations. Populations separated by more than 1,220 km still present a common signal (asymptotic rho = 0.12±0.004, Fig 3). This non-linear, negative relationship is similarly observed at all spatial scales. Fig 3 also illustrates that coherence over distance is much higher for mean annual temperature chronologies than yearly precipitation chronologies and ring-width signals.

**Fig 4:**
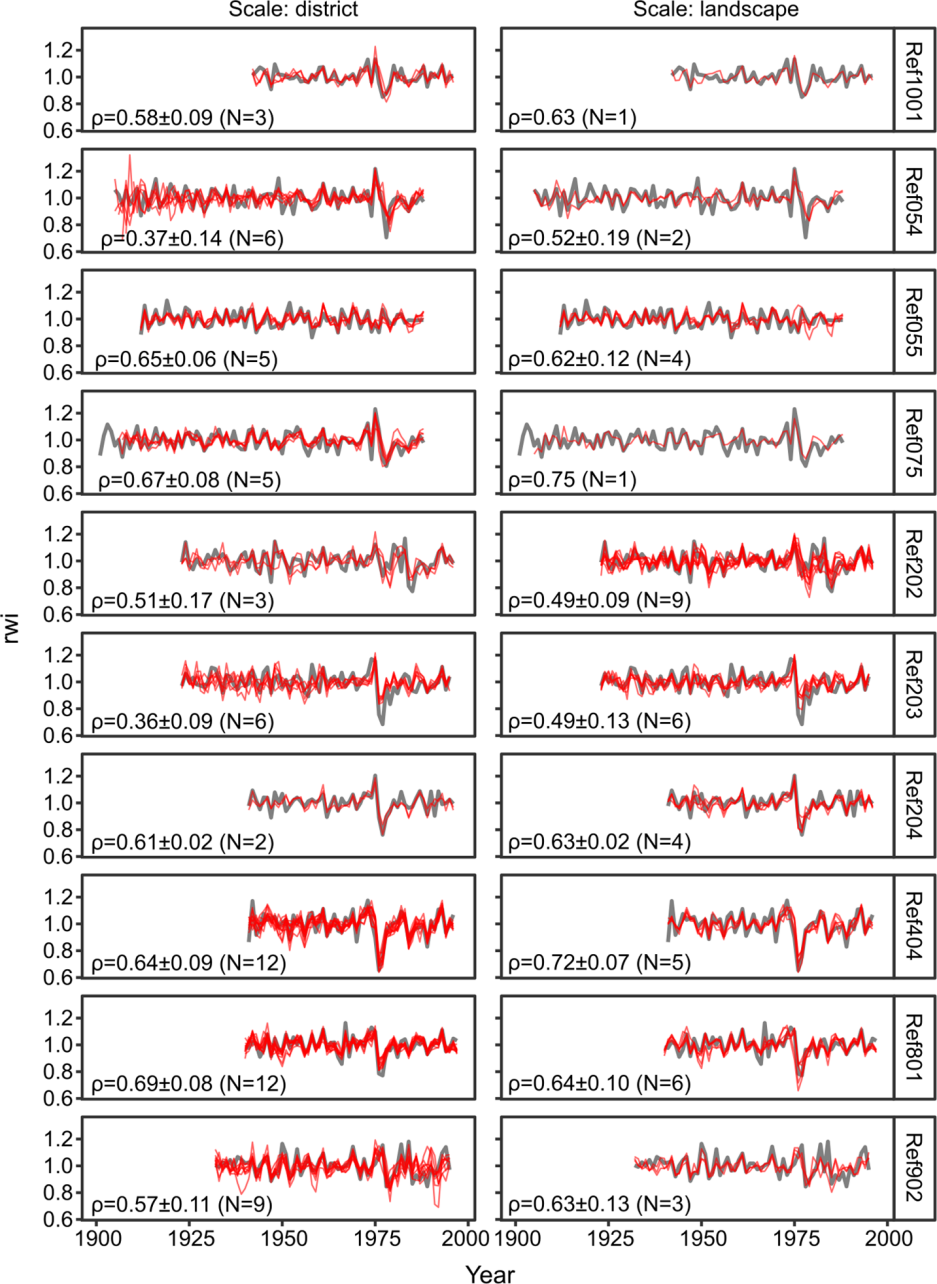
Comparison of the ring-width index (rwi) from ten crossdated reference chronologies (grey) against neighbouring new chronologies (red) built at the district scale (median area: 203 km^2^) and landscape scale (median area: 2,259 km^2^) grouped by soil physical environment type. Only chronologies located within a 40-km radius (district scale) or 80-km radius (landscape scale) of the reference chronologies were used. Radius was determined as the distance between the centroid of the spatial unit and the location of the stand sampled for the reference chronology. Average Spearman correlations (ρ) and associated standard deviations are shown.

Fig 4 summarizes the analysis of correlations between valid master chronologies built at the two finest spatial scales (district and landscape) and neighbouring reference chronologies built at the stand scale. The interannual growth variations of the newly formed chronologies are well synchronized with reference chronologies at both spatial scales. On average, Spearman’s correlation coefficients between newly formed chronologies and reference chronologies ranged from 0.37 to 0.69 for the chronologies built at the district scale, while they ranged from 0.52 to 0.75 for the chronologies built at the landscape scale (Fig 4). For seven out of ten reference chronologies, the fit with the reference chronology was slightly better with chronologies built at the landscape scale as compared to chronologies built at the finest spatial scale (ecological district).

### Probability of false-positive selection and possible hidden signals

**Fig 5:**
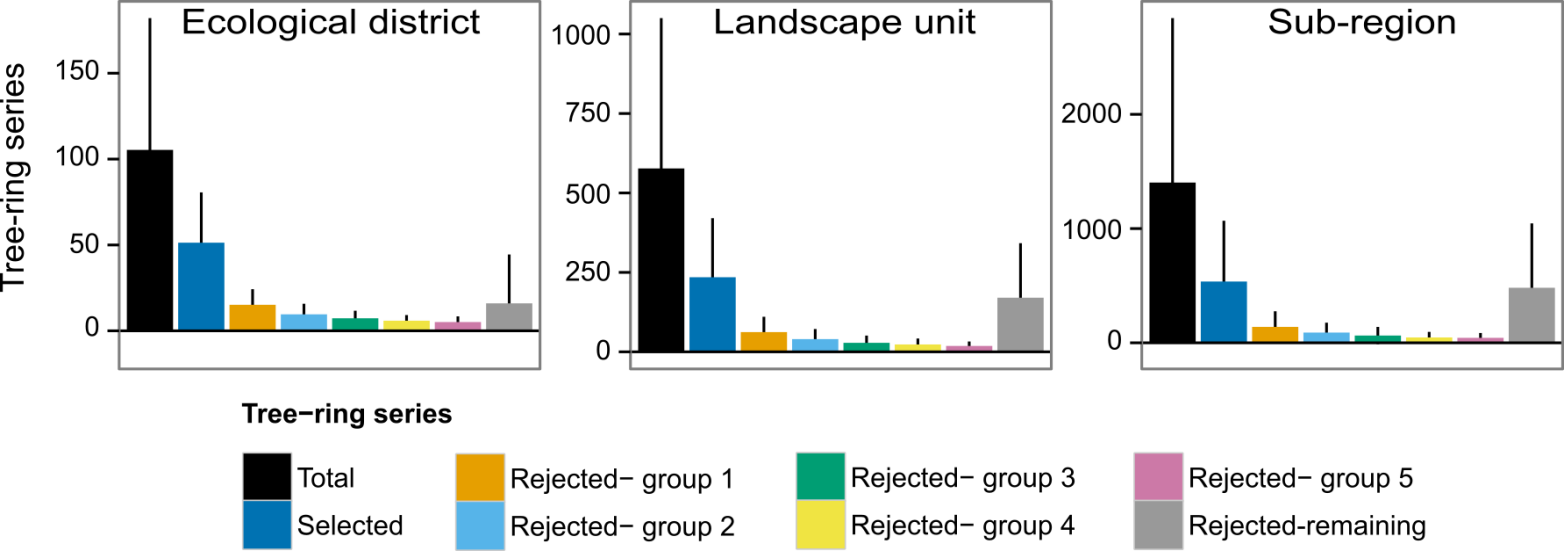
Average number of residual tree-ring series per spatial unit for ecological districts, landscape units, and sub-regions (in black). In addition to the average number of selected series per spatial unit (deep blue), the figure also displays groups of significantly correlated series when selected series are excluded and the filtering procedure is repeated on remaining data, while the grey bar represents the number of remaining series after five iterations of the filtering procedure. Error bars are standard deviations from the means.

First-order autocorrelation of individual standardized series was, on average (±1 SE) 0.0176±0.0003 revealing a low probability of false-positive selection associated to the remaining autocorrelative structure in standardized series. Our analysis revealed that the chronologies formed with the selection procedure represent the largest fraction of the trees sharing a common signal and that no coherent signal may be identified among a large part of the rejected series (Fig 5). The computation of correlations between rejected residual series and the retained chronology for each spatial scale confirmed that >80% of rejected residual series are significantly correlated to the retained chronology at P<0.05 when lagged by one to five years.

**Fig 6:**
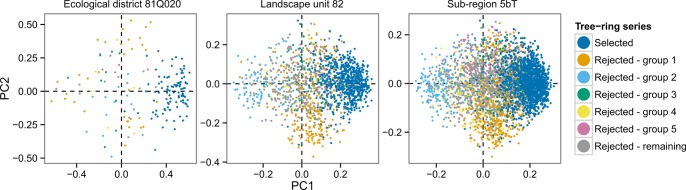
Principal component analysis biplots (scaling type 2) of residual tree-ring series for the most abundant spatial units at the district, landscape, and sub-region scale. Principal components 1 and 2 are displayed. Selected residual series are in deep blue and located at one end of PC1, while other colours represent significantly correlated groups of rejected series when selected series were excluded and the filtering procedure was repeated on remaining data. Lastly, the grey dots represent the remaining rejected series after five iterations of the filtering procedure.

Principal component analysis also revealed that selected series among each group exhibit a common dominant signal across the samples (Fig 6) and confirmed that the rejected series do not share common “hidden” signals. As mentioned earlier, our analysis rather suggests that a vast majority of the rejected series are simply incorrectly dated. Consequently, their signals were asynchronous and incoherent with the population signal.

**Fig 7:**
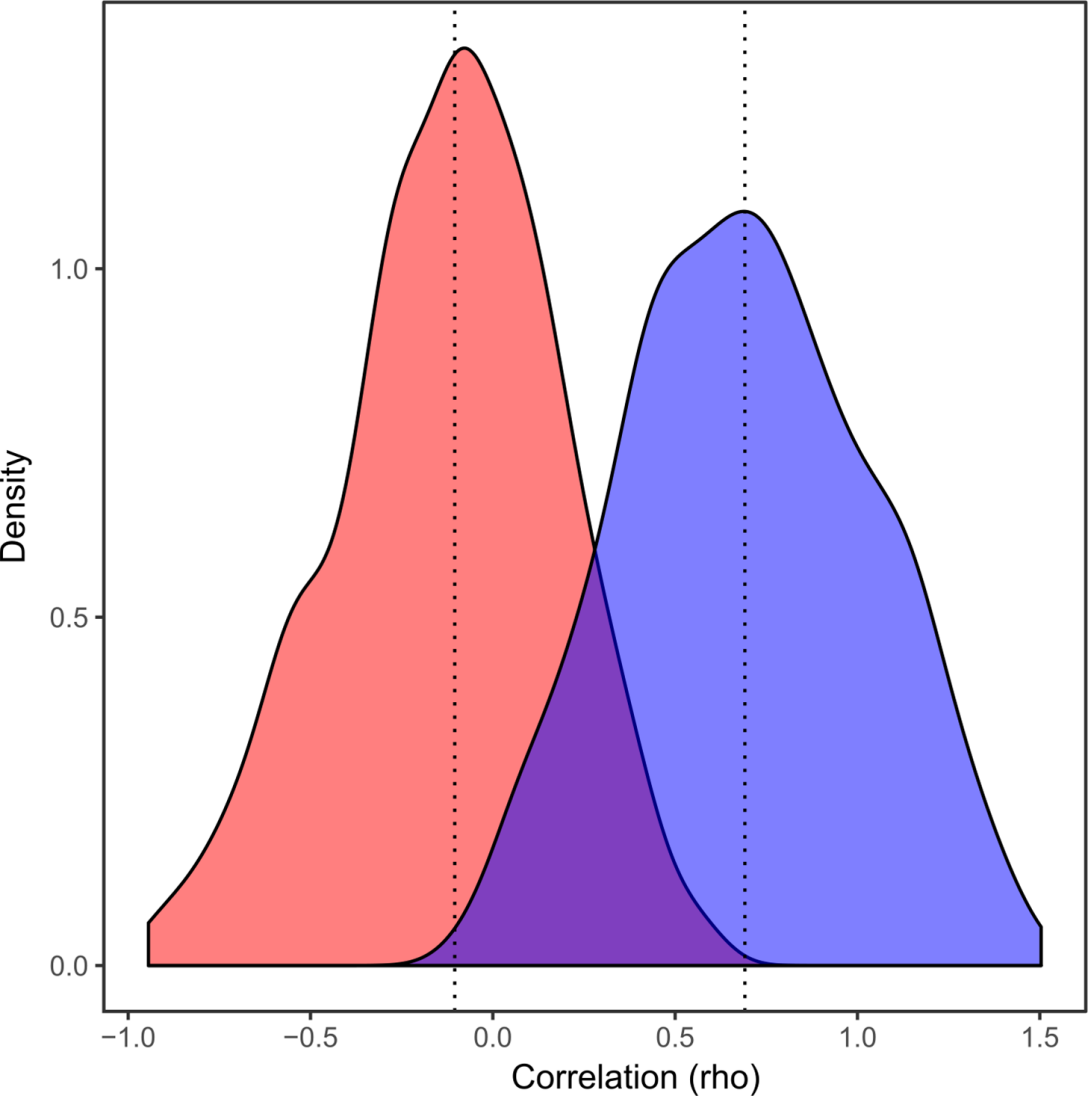
Distribution of segmental coefficients of correlation between rejected series and their respective landscape chronologies at the point of dating (red area, r = -0.12 ± 0.01), and at position (from 5 years earlier to 5 years later) which correlated higher with landscape chronologies than where it has been originally dated (blue area, r = 0.70 ± 0.01). Segment tested are 22 years long (1944–1965, 1955–1976, 1966–1987) restricting analyses to the 1944–1987 period. Dotted lines illustrate median values.

Segmental crossdating analyses of rejected series against their respective regional chronologies revealed that low correlations between the rejected series and regional chronologies (mean *Pearson* coefficient (± 1 SE) = -0.12 ± 0.01) can be improved considerably (mean *Pearson* coefficient (± 1 SE) = 0.70 ± 0.01) by selecting segments which correlated higher at some position other than where it has been originally dated (Fig 7).

## Discussion

### Reliability of the computed chronologies

The strong spatial autocorrelation among independently-built chronologies as well as their high coherence with reference chronologies built at the stand scales shows that coherent interannual growth signals can be extracted from NFI increment core samples at various spatial scales (see next section). Our first hypothesis was confirmed as the strength (Mcor and Rbar) of valid black spruce tree-ring signals (EPS ≥ 0.85) decreased from the local to broad scale (Table 3). The mean correlation of individual series to their master chronologies (Mcor) is nevertheless only slightly related to the spatial dispersion of the samples forming the chronologies. The Mcor statistic is indeed higher at finer spatial scales of sample agglomeration, but decreases exponentially as sample dispersion increases. These results suggest that broad-scale climate drivers partly regulate interannual tree growth variations over a broad spatial scale, but that locally, coherence in tree-ring chronologies is higher as they integrate tree response to local disturbances and smaller-scale climatic variability. Variability in growth response to climate due to tree’s genetic origin may also play a role in the observed pattern.

Accordingly, the proportion of individual series showing a coherent signal and retained by the selection procedure also decreased as sample dispersion increased. At the finest spatial scale studied (ecological district, median area 228 km^2^), nearly two thirds of the individual series (65.8%) from the large majority of the sites (84.6%) showed coherent tree-ring signals. This contrasts with the broadest spatial scale where only 35.7% of the series from slightly more than half of the sampled sites (60.7%) showed coherent signals. The lower sample selection ratio associated with broad-scale computations may be associated with the spatial variability of tree growth drivers inducing local or individual tree growth responses that could be asynchronous at broader scales. For example, trees may respond locally to a drought stress or an insect outbreak that blurs the broad-scale climate signal. These chronologies may exhibit high local coherency while being incoherent with the broad-scale signal. Consequently, these series are retained when the selection procedure is applied locally, but rejected by the selection procedure applied over a broad spatial scale. Series may also be rejected because they do not respond to interannual climate variability due to their growing conditions (i.e. suppressed or declining trees). At all spatial scales, however, additional analyses suggest that the vast majority of rejected series were simply incorrectly dated (see following sections).

Our analysis also revealed that constraining the variability associated with soil physical environments when computing chronologies increased the number of sites and trees retained by the selection procedure and slightly improved the strength of the chronologies (Mcor and Rbar). Considering soil physical environment reduces inter-site variability and thus slightly increases inter-tree signal coherence and the reliability of the computed chronologies. Biophysical variables affecting site quality and tree growth also influence inter-tree competition and thus their sensitivity to climate variability. It has been demonstrated, for instance, that soil organic matter thickness might affect black spruce climate–growth relationships [8,49] which is in good agreement with our observation.

Nevertheless, taking into account soil physical environment types for sample stratification inevitably results in lower sample replication within each stratum. Given that the EPS criterion is highly sensitive to sample replication, a higher fraction of the chronologies was thus rejected by the EPS criteria when soil physical environment type was considered (Table 3). Accordingly, comparable sample selection ratios were observed whether or not chronologies with EPS values lower than 0.85 were rejected, except for the finest spatial scale where the selection ratio was considerably lowered by the EPS criteria due to the low sample replication within each stratum.

Spatial variations in climate, coupled with spatial and temporal variability in the disturbance regime, necessarily induce noise in regional chronologies. The spatial scale at which tree-ring signals should be analyzed is based on the study objectives. The weaker coherence in tree-ring signals observed at broad spatial scales is compatible with those of higher within-plot correlations in tree-ring signals compared to between-plot correlations [10]. Although coherence in tree-ring signals is weaker when computed over a broad spatial scale, it nevertheless assigns more weight to commonly shared growth patterns and reduces the emphasis on local, non-climatic factors [9,15].

### Spatial coherence of chronologies

The strong spatial autocorrelation reported in Fig 3 confirms our second hypothesis that spatial coherence among master chronologies decreases with geographical distance. Correlograms of ring width time series compare very well with those of Wettstein et al. [16] who reported correlations between 762 standardized site chronologies (from the ITRDB), distributed throughout the Northern hemisphere as a function of geographic distance. Their analysis revealed that regionally dependent and species-specific ring width–local climate relationships translate into regionally dependent and species-specific correlograms of ring width time series.

Ring width and yearly precipitation chronologies exhibit weaker spatial coherence than mean annual temperature, confirming that standardized ring width time series not only reflect interannual temperature variability, but are also governed by factors related to water regime and various climate events inducing interannual growth variations [31]. Many studies have indeed documented how tree growth at northern latitudes may respond to a variety of climate conditions, including seasonal temperature and precipitation, short-term events such as drought and heat waves, lagged effects from previous year conditions, snowfall, and melt timing (e.g. [31,50,51]).

In addition to the various climatic factors, non-climatic factors (e.g. insect defoliators, inter-tree competition, tree age, and site characteristics) also induce additional spatial variability in standardized ring width time series. A large part of these non-climatic signals was removed by the standardization processes; however, depending on the model flexibility used for standardization, non-climatic signals may partly remain in the ring width time series even after standardization. Accordingly, Wettstein et al. [16] observed that high-pass filtered ring widths series that emphasize year-to-year variability in tree-ring series exhibit higher spatial coherence than low-pass filtered ring width series. We also acknowledge that errors associated with ring width measurements and dating also induce noise and contribute to weakening the spatial coherence in ring width time series. Researchers should thus be cautioned that all the factors mentioned above, alone or in combination, can reduce the spatial coherence of growth signals as compared to mean annual temperature chronologies.

Master chronologies built at the two finest spatial scales (district and landscape) are also coherent and very well synchronized with neighbouring classic reference chronologies built at the stand scale (Fig 4). Notably, the marked, but spatially asynchronous, growth decrease of black spruce trees at the end of the 1970s in response to the last spruce budworm outbreak is clearly visible in many chronologies and the signal is well synchronized with the reference chronologies [52]. Interannual growth variations of lower amplitude that were presumably associated with interannual climate variability were also very well synchronized with the signals of the reference chronologies.

### Factors limiting use of chronologies for extracting climate signals

Overall, many series were rejected for every spatial scales studied because their signals were incoherent with the master chronology (Table 3). Many reasons may explain the incoherence with the master chronology. At the stand level, inter-tree competition and edaphic properties, known to influence growth responsiveness to climate [8,53,54], and local small-scale disturbances (e.g. partial windthrow, partial cutting, and low to moderate insect defoliation) may induce specific tree-ring signals that are incoherent with the average signal identified at broader spatial scales. Spatial variability in climate and microclimate may also induce specific regional tree growth responses that differ from the broad-scale signal. Accordingly, over broad spatial scales, fewer trees showed coherent signals with the master chronologies and were retained by our selection procedure as compared to local compilations (Table 3).

The selection procedure was also slightly biased toward the rejection of young trees, presumably because their chronologies were not long enough to be statistically correlated with the broad-scale signal of the average chronology or because of a divergent growth–climate relationship between young and old trees [55]. There is indeed evidence that trees at young growth stages are driven primarily by inter-tree competition for light and much less by climate variation, such that climate signals are reduced [53]. An alternative but not mutually exclusive hypothesis is that young fast growing trees growing in open stand conditions are less sensitive to climate variability compared to older trees.

Missing and false rings are generally perceived as a common source of error in tree-ring research. In certain years characterized by unfavorable growth conditions, a tree may not develop an annual ring (missing ring) because of a lack of cambial activity [46]. However, such behavior is rather uncommon in temperate and boreal trees [56]. The formed rings can also be very thin (microrings) and hardly detectable or they may be lacking at some point on the tree (locally absent) and consequently undetectable in a core sample. In addition, specific climatic conditions during the growing season may translate into the formation of a false ring (or double ring) [57]. Missing and false rings can only be identified and located by crossdating. Chronological statistics – coupled with the high correlation among independently-built NFI chronologies and with reference chronologies built at the stand scale – nevertheless suggest that these growth anomalies do not have significant impacts on the quality of the NFI chronologies. The high sample replication and their wide distribution reduce the likelihood that all trees within a population will exhibit a synchronous missing or false ring.

The low residual first-order autocorrelation of individual standardized series revealed a low probability of false-positive selection associated to the remaining autocorrelative structure in standardized series. The computation of correlations between rejected residual series and the retained chronology confirmed that a large part of the rejected residual series are significantly correlated to the retained chronology when lagged by one to five years. In addition, segmental crossdating analyses of rejected series against their respective regional chronologies revealed that series crossdating can be improved considerably by selecting segments which correlated higher at some position (from 5 years earlier to 5 years later) other than where it has been originally dated. Such results suggest that a vast majority of rejected series are simply incorrectly dated and that crossdating of many rejected series may have been improved by implementing a quality control procedure to validate tree ring measurements and dating during sample processing.

A growing number of studies are revealing that there is a large underappreciated problem with tree ring data, which is that tree ring sampling procedures may lead to spurious growth trends as sampled trees are not representative of the entire cohort of trees that lived in the past [20,58–60]. This is particularly apparent in tree ring studies, where only living, dominant and co-dominant trees are sampled [20,58,59]. Systematic and relatively large sampling biases in tree ring studies are practically unavoidable. Just like the crossdating approach, our selection procedure is inevitably biased toward the selection of trees showing a common signal rejecting trees exhibiting incoherent signals. This has no implication for studies aiming to reconstruct high frequency historical climate variation from tree ring, but it may lead to biased interpretations and conclusions for studies documenting species-specific growth response to climate variability as growth-climate correlations would be overestimated in comparison with the entire tree population in a region. As a general statement, we therefore recommend great caution in interpreting results from tree ring studies and caution that sampled trees are rarely representative of the entire population.

## Conclusion

In this study, we developed a procedure to select valid individual tree-ring width time series from an extensive collection of ring width measurements from nearly 94,000 black spruce trees distributed over a 500,000 km^2^ area and collected as part of the NFI in Quebec, Canada. Our results confirm that coherent signals may be extracted from large, raw increment core measurements and used for dendroclimatic investigations. Although signal coherency decreases with geographical distance between sample sites, the remaining signal is representative of large regional drivers such as climate. We thus conclude that tree-ring data from NFIs provide an extraordinary opportunity to strengthen the spatial coverage of tree-ring data in terms of climate zone, species composition, and forest productivity, and to improve coordination with other contemporary measurements of forest growth in order to provide a better understanding of tree growth–climate relationships over broad spatial scales.
